# Severe Periodontitis and Biomarkers of Bacterial Burden. Results From a Case-Control and Intervention Clinical Trial

**DOI:** 10.3389/froh.2021.615579

**Published:** 2021-03-05

**Authors:** Yago Leira, Dimitrios Fragkiskos, Marco Orlandi, Jeanie Suvan, Luigi Nibali, Maurizio S. Tonetti, Georgios N. Belibasakis, Nagihan Bostanci, Francesco D'Aiuto

**Affiliations:** ^1^Periodontology Unit, UCL Eastman Dental Institute and NIHR UCLH Biomedical Research Center, University College London, London, United Kingdom; ^2^Medical-Surgical Dentistry (OMEQUI) Research Group, Health Research Institute of Santiago de Compostela, Santiago de Compostela, Spain; ^3^Periodontology Unit, Center for Host Microbiome Interactions, Faculty of Dentistry, Oral & Craniofacial Sciences, King's College London, London, United Kingdom; ^4^Division of Periodontology and Implant Dentistry, Faculty of Dentistry, Prince Philip Dental Hospital, University of Hong Kong, Hong Kong, China; ^5^European Research Group on Periodontology, Genova, Italy; ^6^Division of Oral Diseases, Department of Dental Medicine, Karolinska Institutet, Huddinge, Sweden

**Keywords:** periodontitis, soluble triggering receptor expressed on myeloid cells 1, bacterial endotoxin, biomarkers, treatment

## Abstract

**Background and aims:** Periodontitis is an inflammatory-infectious disease. Identifying markers of systemic exposure of periodontitis might be of interest to study its interaction with other conditions. Soluble triggering receptor expressed on myeloid cells 1 (sTREM-1) is upregulated during bacterial infections. Our aim was therefore to investigate whether periodontitis and its treatment are associated with bacterial endotoxin and sTREM-1.

**Methods:** Fifty patients with severe periodontitis and 50 age-matched controls were included in a case-control study (all never smokers). A secondary analysis of a previously published intervention study was performed, in which included 69 patients with severe periodontitis were randomized to receive either intensive (IPT) or control periodontal therapy (CPT) and monitored over 6 months. Serum levels of bacterial endotoxin and sTREM-1 were determined at one time point (case-control study) and at baseline, 1 day, 1 and 6 months after periodontal treatment (intervention study).

**Results:** Severe periodontitis was associated with elevated circulating endotoxin levels when cases (22.9 ± 2.2 EU/ml) were compared to controls (3.6 ± 0.5 EU/ml, *p* < 0.001) and with sTREM-1 levels (1302.6 ± 47.8 vs. 870.6 ± 62.0 pg/ml, *p* < 0.001). A positive correlation was observed between sTREM-1 and endotoxin levels (*r* = 0.4, *p* < 0.001). At 6 months after treatment, IPT significantly decreased serum levels of sTREM-1 compared to CPT (adjusted mean difference of 500.2 pg/ml, 95% CI: 18.9–981.4; *p* = 0.042). No substantial differences were noted in endotoxin levels at any time point after treatment between groups.

**Conclusions:** Severe periodontitis is linked to increased circulating endotoxin and sTREM-1 levels and following IPT a reduction in sTREM-1 levels is observed.

## Introduction

Triggering receptor expressed on myeloid cells 1 (TREM-1) is a transmembrane glycoprotein that is involved in the amplification of proinflammatory responses after bacterial and fungal infection [1–3]. TREM-1 expression is upregulated in response to bacterial endotoxin (e.g. lipopolysaccharide) and together with toll-like receptors activation results in increased production of pro-inflammatory mediators, neutrophil degranulation and macrophage phagocytosis [3–6].

Periodontitis is a chronic inflammatory disease triggered by a dysbiotic dento-gingival biofilm [7]. A number of local and systemic biomarkers have been studied to examine the host response both at the gingival and systemic level [8, 9]. Release of the soluble form TREM-1 (sTREM-1) has been demonstrated in body fluids such as saliva [10, 11], gingival crevicular fluid (GCF) [12–14] and peripheral blood (e.g., serum) [10]. This seems to be regulated by the engagement to its putative ligand peptidoglycan recognition protein 1 (PGLYRP1) and the proteolytic cleavage by matrix metalloproteinases or bacterial proteases [15–17].

Similarly, there is an overexpression of mRNA TREM-1 in gingival tissues presenting with signs of inflammation [18]. Observational evidence mainly derived from cross-sectional studies suggests that patients with periodontitis exhibit increased levels of sTREM-1 both in oral fluids (saliva and GCF) [10–14] and serum [10] when compared to individuals with a healthy periodontium. However, many confounders could explain this association, that is, aging [19] or presence of systemic disease [20]. On the other hand, microbiological evidence suggests that sTREM-1 also correlates with high bacterial load of putative periodontal pathogens such as *Porphyromonas gingivalis* (*Pg*), *Treponema denticola* or *Tannerella forsythia* [13]. *Pg* triggers sTREM-1 release from monocytic cells and neutrophils [17, 21]. Of particular interest is the use of subantimicrobial doses of doxycycline seems to abrogate these effects [16].

Based on its ability to increase under both infectious and non-infectious inflammatory conditions [1, 22–24], sTREM-1 could represent a useful biomarker of systemic exposure in chronic diseases like periodontitis. Indeed in periodontitis, it has been shown that endotoxins from *Pg* can inactivate immune cells responses to other oral bacteria, thus, facilitating the development of the typical chronic inflammatory lesion seen in periodontitis [25]. On the other hand, while acute exposure to endotoxins from bacterial origin can lead to a hypercoagulable state [26] and sepsis [27], chronic exposure to endotoxins can be associated with chronic inflammatory conditions such as atherosclerotic vascular diseases [28] or metabolic syndrome [29].

Previously, our group reported that effective control of periodontal infection (i.e., intense supragingival tooth cleaning with root subgingival debridement with or without the use of local antimicrobials) might have an impact systemically reducing systemic inflammation [9, 30], improving blood vessels function [31] as well as helping to maintain a better glycaemic control [32]. It would be of interest to test whether this intense periodontal therapy (IPT) has an impact on systemic levels of biomarkers of bacterial burden compared to a control periodontal therapy (CPT) in which only supragingival scaling and polishing is performed.

We hypothesize a correlation between sTREM-1 and circulating endotoxin levels in patients with severe periodontitis when compared to controls and possible effect of periodontal treatment on these biomarkers. The purpose of this study was, therefore, two-fold. Firstly, we aimed to compare serum levels of bacterial endotoxin and sTREM-1 in patients with severe periodontitis and compared to age-matched controls (case-control study). Secondly, we explored the effect of periodontal therapy on these biomarkers in a secondary analysis from a randomized controlled clinical trial with 6-months follow-up (intervention study).

## Methods

### Case-Control Study

We selected cases and controls from a large observational study completed at UCL Eastman Dental Institute and Hospital between 2005 and 2011 (nested case-control) [33]. The cases consisted of 50 patients with severe periodontitis [including both cases with earlier definition of aggressive [34] and/or chronic periodontitis with at least 50% of teeth with deep pockets of >6 mm showing >30% of radiographic bone loss] [30]. Radiographic bone loss (>1/3 of the root length) was calculated on existing radiographs collected during the routine screening examinations (either panoramic tomography or periapical assessment). Fifty age-matched (1:1 ratio) healthy controls without any clinical/radiographic signs of periodontitis or history of disease were recruited among referrals to other departments of the same hospital. Exclusion criteria for both groups were as follows: (i) younger than 25 years of age; (ii) <20 teeth present; (iii) former/current smokers; (iv) any concomitant medical conditions (e.g., diabetes) or active infectious diseases (e.g., HIV, hepatitis, tuberculosis); (v) pregnancy or breastfeeding; (vi) prolonged use of medication known to affect host response (e.g., immunosuppressive therapy); (vii) use of systemic antibiotics within 3 months before periodontal examination.

A full-mouth periodontal examination was performed in all participants by calibrated periodontists as previously reported [33]. Briefly, a calibration exercise between the two study examiners was performed on 10 non-study patients. Each examiner showed 99% agreement for measures of clinical attachment level (CAL) within 2 mm of difference, the intraclass correlation coefficient for CAL was above 0.90 for both (standard deviation between the two measures equal to 1.05) and the inter-examiner correlation was >0.80. The following parameters were measured at all teeth (except third molars): probing pocket depth (PPD), CAL, full-mouth plaque score (FMPS), and full-mouth bleeding score (FMBS) [35]. Measurements were recorded at six sites per tooth (mesio-buccal, disto-buccal, mid-buccal, mesio-palatal/lingual, disto-palatal/lingual and, mid-palatal/lingual), using a calibrated University of North Carolina periodontal probe (UNC 15; Hu-Friedy, Chicago, IL, USA) with a light probing force of 0.3 N. The patient's height (m) and weight (kg) were recorded and then body mass index (BMI) was calculated using the formula (weight/height^2^, kg/m^2^). A blood sample was taken by trained staff. The average systolic and diastolic blood pressures (SBP and DBP, mmHg) were calculated from three separate readings of the same designated arm and recorded by a trained nurse. Ethnic origin was confirmed by a questionnaire.

This study was approved by the human local ethics committee (ref no. 05/Q0502/84) and all participants provided written informed consent. The study was conducted in accordance with the Helsinki Declaration of 1975, as revised in 2013. STROBE (STrengthening the Reporting of OBservational studies in Epidemiology) guidelines were followed to perform this research [36].

### Intervention Study

Data and sufficient serum samples were obtained from 69 patients with severe periodontitis (at 50% of teeth with pockets of >6 mm and alveolar bone loss of >30%) [30] who participated in a 6-month randomized clinical trial previously reported (Trial ID: NCT00327561), which took place at UCL Eastman Dental Institute and Hospital between 2000 and 2006 [31]. One calibrated periodontist masked to treatment assignment performed full-mouth periodontal assessments in all participants at baseline and 6 months [37]. In brief, a total of 10 non-study subjects were recruited and used for calibration of the examiner. These subjects had periodontal disease and the examiner recorded in two different occasions full mouth PPD and gingival recession at six sites per tooth (excluding third molars) using a manual, UNC- 15 periodontal probe. CAL was calculated from PPD and gingival recession. Upon completion of all measurements, intra-examiner repeatability for CAL measurement was assessed. Examiner was judged to be reproducible after meeting a percentage of agreement within ±2 mm between repeated measurements of at least 98%. Socio-demographic and clinical data were recorded in similar fashion of the case-control study and smoking history was confirmed by a questionnaire. The following exclusion criteria were applied for all study participants: (i) presence of systemic diseases; (ii) previous history of any other acute/chronic infection; (iii) consumption of systemic antibiotic within the 3 months prior to the trial or any other regular medication.

Participants in this study were randomized to either IPT (*N* = 35) or CPT (*N* = 34) as previously described [31]. Briefly, before treatment all patients received basic oral hygiene instructions. After extraction of unsalvageable teeth, participants in the IPT group underwent one single session of supra and subgingival tooth cleaning of all dentition under local anesthesia with no limitation of time and at the end of the session, microspheres of minocycline (Arestin®, OraPharma, Warminster, PA, USA) were applied locally into the periodontal pockets. In the CPT group, full-mouth supragingival cleaning and polishing was performed in a single session. Serial blood samples were collected and available for analysis at baseline, 1-day, 1 and 6 months after completion of therapy. All patients gave written informed consent and the study was approved by the human local ethics committee (ref no. 03/E007) and was conducted in accordance with the Helsinki Declaration of 1975.

### Biomarkers Assessment

Fasting (overnight) blood samples were collected in patients from both studies and processed within 1 h of collection. Centrifugation at 3,000 rpm for 15 min allowed storage of multiple aliquots of 1 mL in a −70°C freezer until analysis. Serum endotoxin activity was determined by the limulus amebocyte lysate (LAL) QCL-1000 high sensitivity assay kit (Lonza, Walkersville, MD) with a chromogenic substrate according to manufacturers' instructions. Samples were diluted (1:5, vol/vol in endotoxin-free water) if the concentration of endotoxin in the test sample was >1.0 EU/ml. A high sensitivity DuoSet Enzyme-Linked ImmunoSorbent Assay (ELISA) kit (R&D Systems, Abingdon, UK) was used to analyse serum levels of sTREM-1 according to manufacturers' instructions. Intra and inter-assays coefficients of variation for both assays were <7%. To minimize variability, all assays were performed at the end of the study, in duplicates and with the same kits at both time points by staff masked to study and treatment allocation.

### Statistical Analysis

A sample size of 48 patients per group (case vs. controls) (1:1 ratio) was sufficient to detect a 200 pg/ml difference in serum sTREM-1 between study groups, with a standard deviation of 300 pg/ml and assuming α-value = 0.05 and power of 90% [10].

No formal sample size calculation was performed in the intervention study (a *post-hoc* power analysis based on the results obtained from the study confirmed a 80% power to detect a 50% reduction in sTREM-1 but only 10% power to detect 20% reduction in bacterial endotoxin).

Mean values and standard deviation were calculated for continuous variables. Categorical data are reported as percentages (%) and compared by McNemar's test. Average values of biomarkers in the case-control study were compared using one-way analysis of variance (ANOVA). The primary outcome for this study was sTREM-1 values and as covariates we considered gender, BMI and ethnicity. Univariate analyses were performed between each biomarker and all other oral and systemic variables collected at the visit. Those variables with a statistical significant association with the primary outcome (sTREM-1) were included in to a multivariate model. Multiple linear regression models were fitted for bacterial endotoxin and sTREM-1 including the following covariates: gender, ethnicity and BMI. Log-transformation was applied when normality assumptions were not met. Non-parametric correlation analyses between clinical periodontal parameters and biomarkers were performed using Spearman rank correlation coefficient (r).

In the second study (intervention trial), we performed a secondary analysis of samples obtained from a previous published randomized clinical trial and the primary outcome was the difference in sTREM-1 levels between study groups at 6 months using analysis of covariance (ANCOVA). Changes in bacterial endotoxin at 6 months after treatment was a secondary outcome and a number of covariates were included in the model: age, gender, smoking, ethnicity and BMI (following univariate associations as described earlier). In addition ANOVA for repeated measure models were fitted to compare the changes of both biomarkers between study groups at 1 day, 1 and 6 months using the following covariates: age, gender, smoking, ethnicity and BMI. Due to the limited number of never smokers available in this secondary analysis we decided to include both smokers and not in the final analysis. A sensitivity analysis was however performed by repeating the analysis in the subgroup of never smokers and those with current/former smoking history.

All tests were performed at a significance level of α = 0.05 using either STATA (ver 14) (StataCorp LLC, Texas, USA) or SPSS Statistics (version 24.0) (SPSS Inc., Chicago, IL, USA).

## Results

### Case-Control Study

Cases and controls were balanced for gender but with a greater prevalence of Caucasians (*p* < 0.001) (Table 1). Cases presented with higher values of office arterial blood pressure (both SBP and DBP), BMI and all clinical indicators of periodontal health (*p* < 0.001) except for dental plaque levels.

**Table 1:** Participants' characteristics at baseline (case-control study, *N* = 100).

**Variable**	**Controls (*N* = 50)**	**Cases (*N* = 50)**	***P*-value**
Age, Years	43 ± 9	43 ± 9	1
Gender, Male (%)	25 (50)	16 (32)	0.103
Ethnicity, Caucasian (%)	50 (100)	13 (26)	**<0.001**
BMI, kg/m^2^	23.7 ± 3.4	27.3 ± 10.5	**0.023**
SBP, mmHg	120 ± 12	128 ± 15	**0.005**
DBP, mmHg	76 ± 8	83 ± 12	**0.001**
FMPS, %	49.9 ± 22.7	45.1 ± 26.8	0.337
FMBS, %	21.9 ± 13.4	55.0 ± 23.4	**<0.001**
Average PPD, mm	1.98 ± 0.24	3.80 ± 1.15	**<0.001**
Average CAL, mm	1.99 ± 0.25	4.12 ± 1.24	**<0.001**

*BMI, body mass index; SBP, systolic blood pressure; DBP, diastolic blood pressure; FMPS, full-mouth plaque score; FMBS, full-mouth bleeding score; PPD, probing pocket depth; CAL, clinical attachment level. Significant results are reported in bold*.

**Figure 1:**
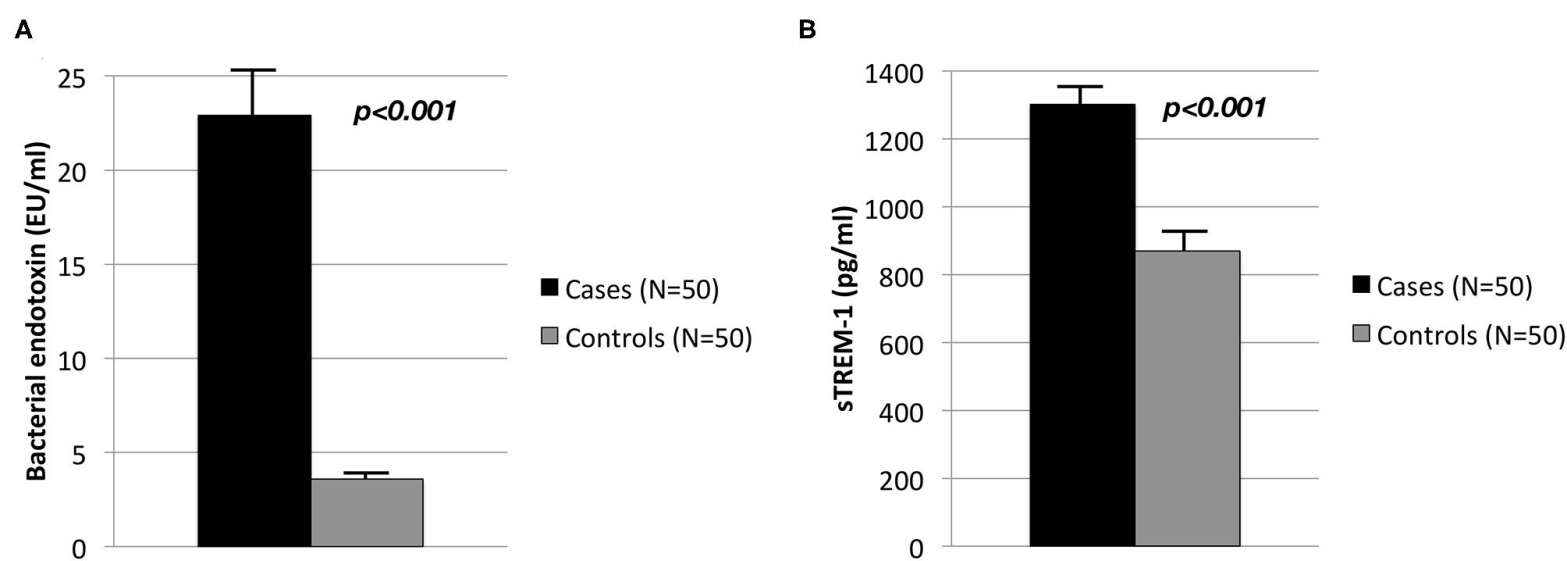
Circulating levels in controls (*N* = 50) and cases (*N* = 50) of **(A)** bacterial endotoxin (EU/ml) and **(B)** sTREM-1 (pg/ml).

Adjusted Circulating levels of bacterial endotoxin were elevated in cases with severe periodontitis when compared to controls (22.9 ± 2.2 EU/ml vs. 3.6 ± 0.5 EU/ml, *p* < 0.001) (Figure 1A). Similarly cases exhibited significantly higher serum levels of sTREM-1 than controls (1,302.6 ± 47.8 pg/ml vs. 870.6 ± 462.0 pg/ml, *p* < 0.001) (Figure 1B).

**Figure 2:**
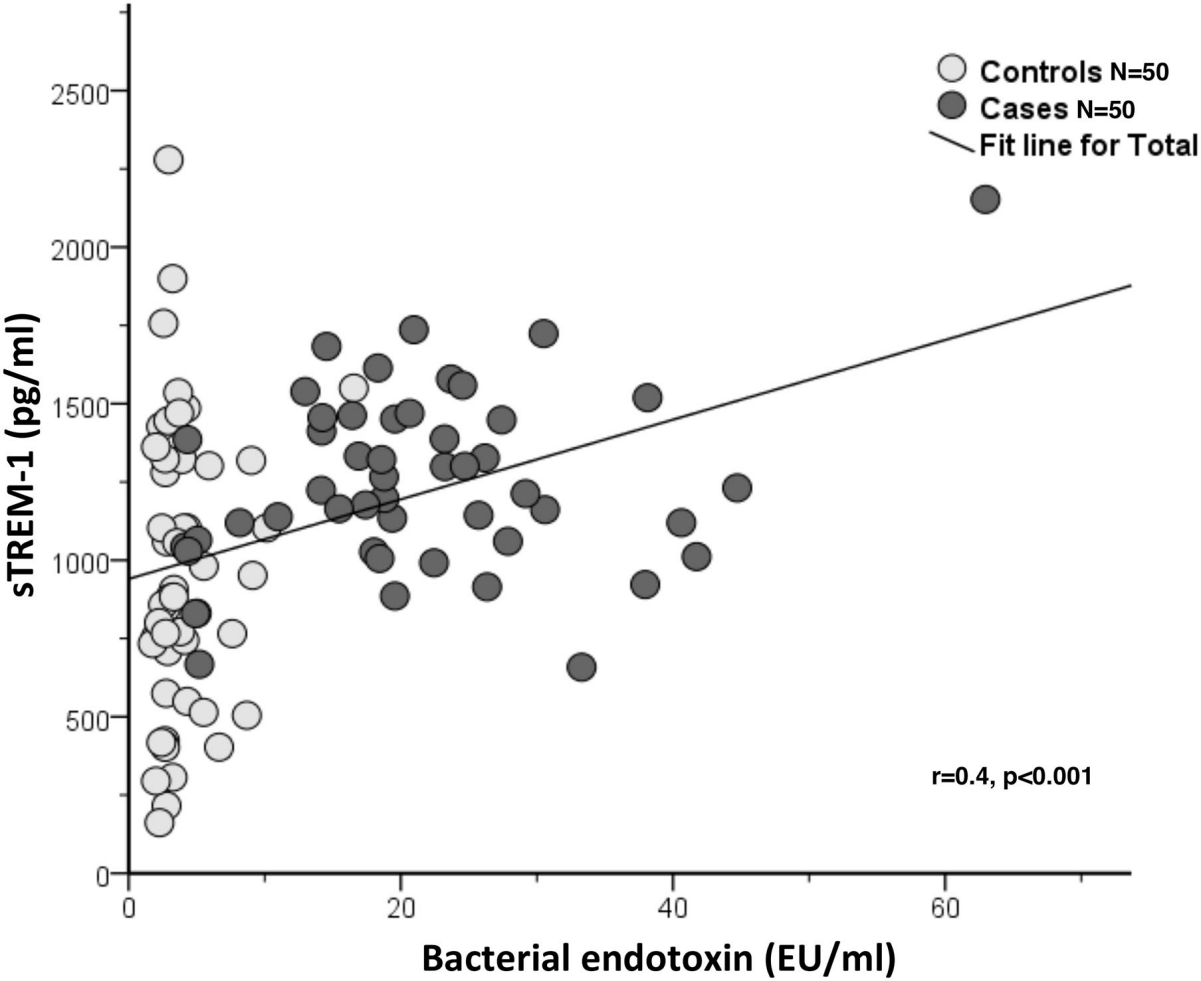
Correlation between sTREM-1 (pg/ml) and bacterial endotoxin (EU/ml) in the case-control study (one time point) (*N* = 100).

A positive linear correlation between circulating endotoxin levels and sTREM-1 with PPDs, CALs and gingival bleeding were observed but not with dental plaque scores (Table 2). A moderate correlation between circulating bacterial endotoxin and sTREM-1 was also observed (*r* = 0.4, *p* < 0.001) (Figure 2).

**Table 2:** Correlation values (*r*) among biomarkers of bacterial burden and clinical periodontal parameters in the case-control study (one time point).

	**PPD**	**CAL**	**FMPS**	**FMBS**
	**(mm)**	**(mm)**	**(%)**	**(%)**
Bacterial endotoxin (EU/ml)	0.7	0.7	−0.1	0.5
*P*-value	**<0.001**	**<0.001**	0.437	**<0.001**
sTREM-1 (pg/ml)	0.5	0.4	0.0	0.4
*P*-value	**<0.001**	**<0.001**	0.790	**<0.001**

*sTREM-1, soluble triggering receptor expressed on myeloid cells-1; FMPS, full-mouth plaque score; FMBS, full-mouth bleeding score; PPD, probing pocket depth; CAL, clinical attachment level. Significant results are reported in bold*.

### Intervention Study

Study participants were slightly older than those recruited in the case-control study, they were similar in gender, ethnicity and BMI differences but presented with substantially greater levels of gingival inflammation, periodontal attachment loss and dental plaque levels (Table 3). IPT produced a substantial reduction of all clinical parameters of gingival inflammation when compared to CPT patients including the number of sites with deeper PPDs (Table 3).

**Table 3:** Participants' characteristics at baseline and 6 months visits (intervention study, *N* = 69).

**Variable**	**Baseline**			**6 Months**		
	**CPT (*N* = 34)**	**IPT (*N* = 35)**	***P*-value**	**CPT (*N* = 34)**	**IPT (*N* = 35)**	***P*-value**
Age, Years	49 ± 6	47 ± 8	0.353	–	–	
Gender, Male (%)	15 (43)	20 (58)	0.826	–	–	
Ethnicity, Caucasian (%)	15 (45)	19 (55)	0.352	–	–	
Smokers (%)	15 (43)	20 (57)	0.518	–	–	
BMI, kg/m^2^	27.5 ± 5.8	27.4 ± 5.2	0.933	27.4 ± 5.8	27.0 ± 5.1	0.749
SBP, mmHg	125 ± 18	125 ± 18	0.961	124 ± 16	122 ± 15	0.697
DBP, mmHg	79 ± 10	80 ± 12	0.560	77 ± 10	78 ± 10	0.738
FMPS, %	68 ± 19	68 ± 20	0.934	48 ± 24	27 ± 18	**<0.0001**
FMBS, %	67 ± 18	66 ± 18	0.889	64 ± 22	26 ± 16	**<0.0001**
PPD, mm	4.7 ± 0.7	4.6 ± 0.8	0.657	4.5 ± 0.9	3.0 ± 0.4	**<0.0001**
PPK, *n*	81 ± 26	82 ± 28	0.914	75 ± 30	13 ± 12	**<0.0001**
Number of teeth, *n*	27 ± 3	27 ± 3	0.299	27 ± 3	27 ± 3	0.299

*BMI, body mass index; SBP, systolic blood pressure; DBP, diastolic blood pressure; FMPS, full-mouth plaque score; FMBS, full-mouth bleeding score; PPD, probing pocket depth; CAL, clinical attachment level; PPK, number of periodontal pockets >4 mm. Significant results are reported in bold*.

**Figure 3:**
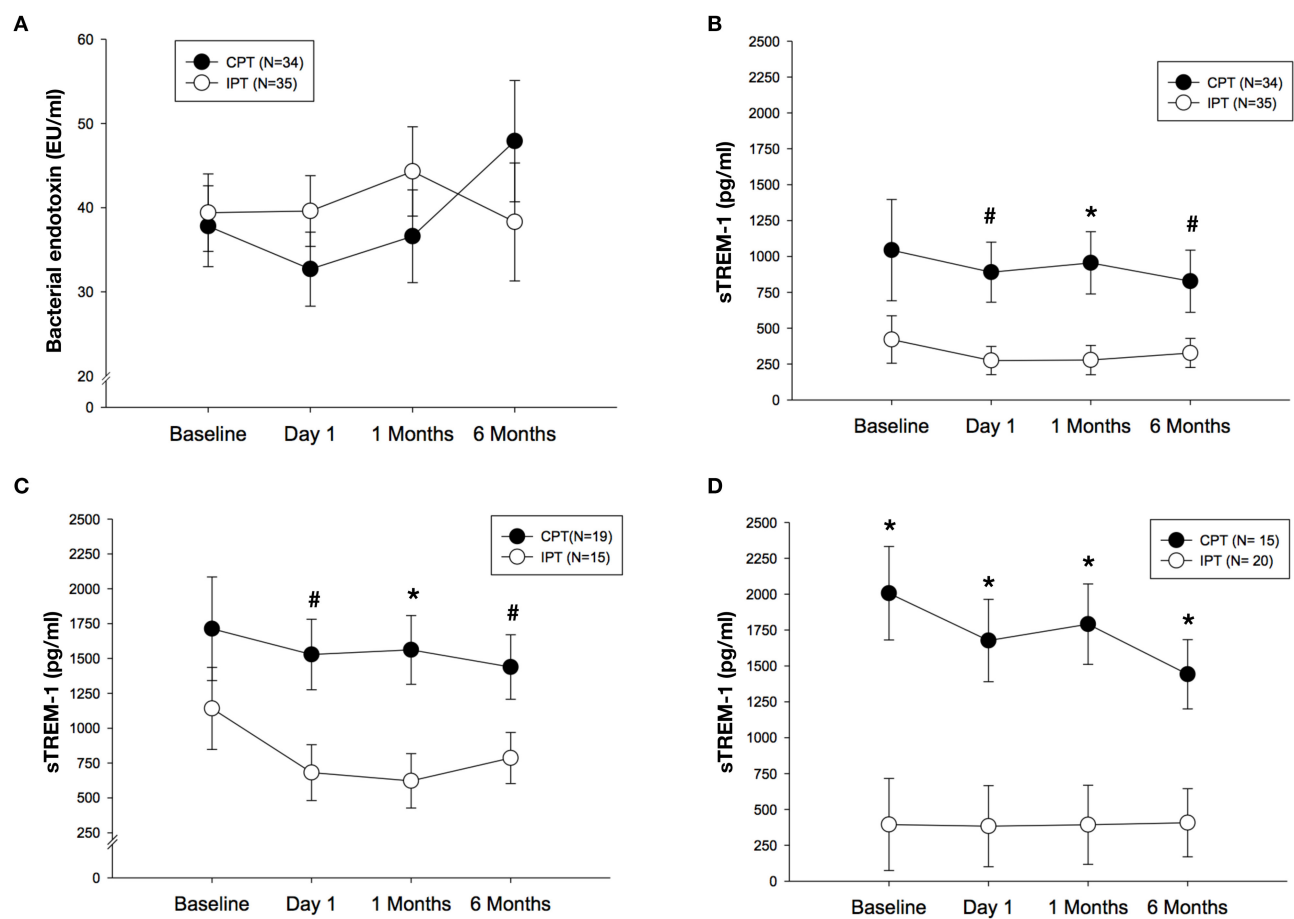
**(A)** Mean (SE) of bacterial endotoxin (EU/ml) after IPT or CPT during the 6 months randomized trial in the whole population. **(B)** Mean (SE) of sTREM-1 (pg/ml) after IPT or CPT during the 6 months randomized study in the whole population. **(C)** Mean (SE) of sTREM-1 (pg/ml) after IPT of CPT in never smokers (*N* = 34). **(D)** Mean (SE) of sTREM-1 (pg/ml) after IPT or CPT in former/current smokers (*N* = 35). #*p* < 0.05 between study groups, *p < 0.01 between study groups.

Serum concentrations of bacterial endotoxin did not differ between groups over time even though values were lower in the IPT group when compared to CPT at 6 months but this difference did not reach statistical significance (adjusted mean difference of 9.6 EU/ml, 95% CI: −10.2, 29.3; *p* = 0.339) (Figure 3A). Circulating levels of sTREM-1 changed over time. Firstly values of sTREM-1 seem to differ between the two study groups at most time points. In adjusted analyses sTREM-1 were greatly reduced in patients undergoing IPT when compared to CPT at 6 months (adjusted mean difference of 500.2 pg/ml, 95% CI: 18.9, 981.4; *p* = 0.042). This effect was noted also after 1 day (adjusted mean difference of 615.8 pg/ml, 95% CI: 151.2, 1,080.4; *p* = 0.010) and 1 month (adjusted mean difference of 677.2 pg/ml, 95% CI: 194.9, 1,159.6; *p* = 0.007) after treatment (Figure 3B).

In never smokers (*n* = 34), differences in sTREM-1 values were more pronounced when IPT patients were compare do those in CPT group [(day 1: adjusted mean difference of 847.0 pg/ml, 95% CI: 184.8, 1,509.2; *p* = 0.014), 1 month (adjusted mean difference of 939.1 pg/ml, 95% CI: 294.5, 1,583.7; *p* = 0.006) and 6 months (adjusted mean difference of 652.6 pg/ml, 95% CI: 47.9, 1,257.2; *p* = 0.035)] (Figure 3C). In those patients who were former or current smokers (*n* = 35), sTREM-1 was different between groups but in the opposite direction compared to never smokers as the values were substantially lower in the IPT group during the study follow-up visits including baseline when compared to CPT at each time point (Figure 3D).

## Discussion

In this study we demonstrated that patients with severe periodontitis had higher circulating levels of bacterial endotoxin and sTREM-1 when compared to age-matched never smokers controls. These two biomarkers correlated positively with measure of active gingival inflammation (PPD and gingival bleeding) and historic periodontal tissue attachment loss. A single-session of non-surgical periodontal therapy combined with locally delivered antimicrobials influenced sTREM-1 levels over 6 months compared to scaling and polishing whilst no effect was observed on serum bacterial endotoxin levels.

The evidence linking periodontitis and bacterial endotoxin levels comes from observational and intervention studies [38–40]. A clear difference in values of endotoxin levels in patients with periodontitis compared to those with a healthy periodontium is confirmed. Further, in this study bacterial endotoxin levels tend to be reduced over the 6 months period after IPT but this difference did not reach statistical significance. This is consistent with the low statistical power to detect a significant difference in the study sample and/or limitations owing to the method of assessment (i.e., short half-life) [41]. Further research using more robust laboratory assays are warranted.

Our results are consistent with previous evidence from observational studies suggesting that oral and systemic sTREM-1 levels are elevated in patients with periodontitis (cross-sectional studies) and correlate with clinical periodontal parameters [10, 12–14, 20]. However, in contrast with previous evidence, in this study no significant correlation was found between measures of dental plaque accumulation and serum sTREM-1 levels [13, 16]. One possible explanation could be that in our study both cases and controls did not different in quantitative measures of dental plaque, it is unknown though whether different microbial composition of the dental biofilm could be responsible for triggering a different systemic host response.

In this study, sTREM-1 highly correlated with circulating endotoxin levels in the whole study sample suggesting it could represent a novel systemic marker of periodontitis [10, 12–14]. Indeed, host exposure to periodontal infection/inflammation is characterized by a systemic inflammatory response which is often quantified by measuring not-specific circulating biomarkers (i.e., C-reactive protein) and there is a clear need of identifying novel biomarkers. This could be relevant especially in patients who suffer from periodontitis and other co-morbidities. Recent evidence suggested that locally produced sTREM-1 is elevated in patients with chronic kidney disease and poor periodontal health confirming an association with local/gingival measures of severity [20].

There is scarce evidence on the effects of periodontal therapy on sTREM-1 [42]. The only trial published confirmed that 1-month after treatment of periodontitis, levels of sTREM-1 in GCF were reduced compared to baseline values [42]. In our study we report different pattern of changes in serum levels of sTREM-1 between patients who received IPT compared to CPT. Indeed we would like to speculate that controlling periodontal infection/inflammation is linked to a reduction of sTREM-1 levels; however differences from baseline values between the two study groups confound the effect of periodontal therapy in our analysis. Further research with the primary aim of confirming/disputing our results should be performed.

Findings from this study revealed that smoking could impact on circulating sTREM-1 levels after periodontal therapy. We observed that in never smokers IPT treatment produced a reduction of sTREM-1 of greater magnitude than current or former smokers. There is conflicting evidence of the effect of cigarettes smoking on circulating sTREM-1 levels [10, 12]. As smoking affects immune cells function, it could also influence the innate immune response to oral resident bacteria [43]. Exposure to cigarettes smoke produces among others changes in macrophage's phenotype [44], reduction in macrophage's phagocytic function [45], alteration of macrophage's survival and genes expression profile [46, 47] or derangement of neutrophil's function [48, 49]. Together these effects make these cells less effective against periodontal bacteria and as a result their ability to produce sTREM-1 could be compromised. As smoking negatively affects the periodontium and the clinical response after periodontal treatment [50], further research is needed in this area to investigate the biological mechanisms underlying a potential relationship between periodontitis and its treatment with sTREM-1 and tobacco consumption.

The present investigation has some limitations. The case-control design of our study does not represent ultimate proof of a causal link between periodontitis and increased peripheral levels of sTREM-1. Matching cases and controls for age and excluding smokers plus the multivariate analyses performed, confirm however the previous evidence suggesting that sTREM-1 is a promising systemic marker of periodontitis. The intervention study is a secondary analysis of a previously completed clinical trial not powered to test the effects of periodontal treatment on sTREM-1 or bacterial endotoxin levels as well as a proportion of study participants were smokers. Whilst we performed a *post-hoc* power analysis, our interpretation of the results urges caution. Future randomized clinical trials with a priori sufficient power and sample size should test the hypothesis that periodontal treatment (non-surgical and possibly surgical) results in changes of systemic sTREM-1 and bacterial endotoxin levels. Lastly, this investigation was focused on patients with severe periodontitis hence our results could not be extrapolated to all patients with different degree of severity and extent of periodontitis. Future case-control and prospective studies should be carried out including milder forms of periodontitis.

In conclusion, severe periodontitis is associated with high circulating levels of bacterial endotoxin and sTREM-1. In addition, periodontitis treatment reduces peripheral levels of sTREM-1.

## Data Availability Statement

The raw data supporting the conclusions of this article will be made available by the authors, without undue reservation.

## Ethics Statement

The studies involving human participants were reviewed and approved by UCL local ethics committee (ref no. 05/Q0502/84 for case-control study and ref no. 03/E007 for intervention study). The patients/participants provided their written informed consent to participate in this study.

## Author Contributions

YL, GB, NB, and FD'A have been involved in conception and study design. DF, MO, JS, LN, and FD'A have been involved in data collection. YL, MT, NB, and FD'A have analyzed and interpreted the data. YL and FD'A have drafted the manuscript. All authors have been involved in revising the manuscript critically and have given final approval of the version to be published.

## Conflict of Interest

The authors declare that the research was conducted in the absence of any commercial or financial relationships that could be construed as a potential conflict of interest.
